# The Role of Education and Verbal Abilities in Altering the Effect of Age-Related Gray Matter Differences on Cognition

**DOI:** 10.1371/journal.pone.0091196

**Published:** 2014-03-13

**Authors:** Jason Steffener, Daniel Barulli, Christian Habeck, Deirdre O’Shea, Qolamreza Razlighi, Yaakov Stern

**Affiliations:** 1 Cognitive Neuroscience Division of the Taub Institute for Research on Alzheimer’s Disease and the Aging Brain; Columbia University College of Physicians and Surgeons, New York, New York, United States of America; 2 Department of Neurology, Columbia University College of Physicians and Surgeons, New York, New York, United States of America; 3 Department of Psychology, Columbia University, New York, New York, United States of America; 4 Department of Psychiatry, Columbia University College of Physicians and Surgeons, New York, New York, United States of America; University of California, San Francisco, United States of America

## Abstract

Evidence suggests that individual variability in lifetime exposures influences how cognitive performance changes with advancing age. Brain maintenance and cognitive reserve are theories meant to account for preserved performance despite advancing age. These theories differ in their causal mechanisms. Brain maintenance predicts more advantageous lifetime exposures will reduce age-related neural differences. Cognitive reserve predicts that lifetime exposures will not directly reduce these differences but minimize their impact on cognitive performance. The present work used moderated-mediation modeling to investigate the contributions of these mechanisms at explaining variability in cognitive performance among a group of 39 healthy younger (mean age (standard deviation) 25.9 (2.92) and 45 healthy older adults (65.2 (2.79)). Cognitive scores were computed using composite measures from three separate domains (speed of processing, fluid reasoning, and memory), while their lifetime exposures were estimated using education and verbal IQ measures. T1-weighted MR images were used to measure cortical thickness and subcortical volumes. Results suggest a stronger role for cognitive reserve mechanisms in explaining age-related cognitive variability: even with age-related reduced gray matter, individuals with greater lifetime exposures could perform better given their quantity of brain measures.

## Introduction

Evidence is accumulating to support the idea that individual variability in lifetime exposures influence how cognitive performance changes with advancing age. Investigations into the mechanisms by which this occurs has led to a number of different theories, see [1] for a recent review. Underlying these theories is the assumption that advancing age leads to brain changes including declines in gray matter [2] and that cognitive performance decline is the result of these neural declines (for a review see [3]). This scheme provides two locations for the effect of lifetime exposures (LE) to operate. Lifetime exposures could influence the effect of advancing age on neural measures. Alternatively, the effect of age-related differences in neural measures on cognitive performance could be influenced by LE differences. These two roles are broadly described in the literature as the theories of brain maintenance (BM) [4] and cognitive reserve (CR) [5], [6].

Brain maintenance hypothesizes that increases in certain LEs can decreases the effect of advancing age on brain integrity as assessed by brain measures [4]. Cognitive reserve focuses on an individual’s usage of their neural tissue, where more efficient or flexible cognitive networks may result in improved, or maintained, performance in the face of neuropathology (be it age-related changes, Alzheimer’s Disease pathology or traumatic brain injury) [7], [8]. In both cases, proxy variables for LE include years of formal education, literacy level, occupational status, engagement in leisure activities and estimated premorbid IQ. Evidence for these proxy variables is based on epidemiologic observations suggesting that these lifetime exposures or abilities reduce the risk of age-related cognitive change or dementia in the face of brain pathology.

Therefore, in exploring how age related neural differences affect cognition, LE may: 1) decrease the effect of advancing age on neural measures supporting the theory of brain maintenance; 2) decrease the effect of age-related differences in neural measures on cognitive abilities, supporting the theory of cognitive reserve; 3) have both of these effects, supporting both theories or 4) have no effect, supporting neither. These four scenarios represent four separate models we tested in this study using moderated-mediation analyses. Moderated-mediation analyses describe an analytical framework testing causal relationships between measures, and whether these relationships are dependent on, or interact with, another variable. These models are statistical path models where each segment of the path is tested using linear regression. Combining the results from each segment the overall path model is tested and significance is assessed using non-parametric statistics. Moderated-mediation analyses are relatively novel to the neuroimaging field [9]–[11]; however, they are well established in the communications field and are an active field of research [12], [13].

Moderated-mediation analyses rely on assumptions of causality. The assumed causal directions of this work are established in the literature. Whole brain volume (WBV) shows a decline of 0.22% per year between the ages of 20 and 80 and accelerates with increasing age [14]. The rates of age-related regional decline in gray matter differ with frontal areas of the brain being especially susceptible to volumetric declines [2]; yet parietal and temporal areas are also highly affected [15]. There is a great deal of research focusing on the relationship between age-related differences in brain volume and cognition. Here we include both of the relationships in a single mediation model, testing whether the relationship between aging and cognition is mediated by differences in brain measures. We can then test whether LS moderates either the relationship between age and gray matter loss, between gray matter loss and cognition, or both. We focus on three well-defined cognitive domains that show age-related differences: episodic memory, speed of processing, and fluid reasoning. These constitute three “reference abilities” designated by Salthouse as capturing the major aspects of age-related cognitive changes [16], [17]. Thus, the current work integrates aging, regional neural measures of subcortical gray matter volume and cortical thickness, the three cognitive domains and a composite measure of lifetime exposures.

## Methods

### Participants

Data from thirty-nine healthy younger (mean age (standard deviation) 25.9 (2.92) and 45 healthy older adults (65.2 (2.79)) were included in this study. Participants were recruited using market-mailing procedures to equalize the recruitment approaches of the two groups. Participants who responded to the mailing were telephone screened to ensure that they met basic inclusion criteria (right handed, English speaking, no psychiatric or neurological disorders, and normal or corrected-to-normal vision). All participants found eligible via the initial telephone screen were further screened in person with structured medical, neurological, psychiatric, and neuropsychological evaluations to ensure that they had no neurological or psychiatric disease or cognitive impairment. The screening procedure included a detailed interview that excluded individuals with a self-reported history of major or unstable medical illness, significant neurological history (e.g. epilepsy, brain tumor, stroke), history of head trauma with loss of consciousness for greater than 5 minutes or history of Axis I psychiatric disorder [18]. Individuals taking psychotropic medications were also excluded. Global cognitive functioning was assessed with the Mattis Dementia Rating Scale, on which a score of at least 133 was required for retention in the study [19]. This study was approved by the Internal Review Board of the College of Physicians and Surgeons of Columbia University and written informed consent was obtained from all participants prior to study participation, and after the nature and risks of the study were explained. Participants were compensated for their participation in the study.

### Composite Measures

Previous factor analyses from our laboratory identified neuropsychological and behavioral measures underlying the construct of lifetime exposure and three cognitive domains: memory, speed/attention and fluid ability [20]. Using these measures, composite scores were created using the mean of the z-transformed measurements. Missing values from any of the measurements were imputed using a simplified version of multivariate imputation based on Principal Components Analysis (PCA) without regard for assumptions such as robustness or the randomness of the missing values. This process estimated the factor scores from the test values present for each participant separately for young and old. None of the missing values were due to a participant’s unwillingness or inability to complete the test, but rather to time constraints during administration and/or experimenter error. Therefore, we believe that the PC structure is the same for those subjects with complete data as those with incomplete data, as is assumed by this procedure. The group means, standard deviations, and correlations are listed in Table 1 and reported with current recommendations of appropriate significant digits [21].

**Table 1:** Composite factors and their measures.

	Young	Old	Correlation Coefficients			
	Mean(s.d)	Mean(s.d.)	Memory	Speed	Fluid	LE
Memory						
SRT Total	56(9.2)	46(8.5)	0.98	0.55	0.67	0.55
SRT Long Term Recall	51(13.6)	34(13.6)	0.98	0.48	0.63	0.48
SRT Delayed Recall	10.1(2.07)	7.26(2.48)	0.94	0.54	0.6	0.54
Speed						
WAIS-R Digit Symbol	64(12.4)	46(12.1)	0.48	0.86	0.62	0.24
Trailmaking Test A	24(11.1)	35(11.4)	−0.41	−0.81	−0.52	−0.33
Stroop Color	79(14.4)	67(12.2)	0.44	0.8	0.49	0.31
Fluid Ability						
WAIS-3 Matrices	19(6.6)	14(6.9)	0.49	0.48	0.82	0.56
WAIS-3 Letter Number	12.7(3.50)	10.2(3.29)	0.53	0.55	0.82	0.45
Block Design	50(11.6)	32(10.4)	0.62	0.63	0.86	0.38
Lifetime Exposures (LE)						
Education	15.6(1.95)	15.4(3.08)	0.31	0.35	0.49	0.75
AMNART errors	15(7.2)	13(11.3)	−0.28	−0.32	−0.45	−0.9
WAIS-R Vocabulary	50(13.8)	54(10.8)	0.28	0.22	0.45	0.86
Cognitive Factors (Z-Scores)						
Memory	.56(.81)	−.48(.83)	–			
Speed	.23(.46)	−.20(.43)	0.54	–		
Fluid Ability	.45(.74)	−.39(.71)	0.66	0.66	–	–
Lifetime Exposures	−.070(.71)	.060(.94)	0.35	0.36	0.55	

Note: Means and standard deviations of the composite scores were computed using the z-scores calculated across age groups.

#### Memory

Memory was defined as the composite score comprising the three sub-scores of the Selective Reminding Task (SRT) – total, delayed recall, delayed recognition [22]. For this task, participants were read a list of 12 words and were asked to recall the words after each of six trials. After each recall attempt, participants were reminded of the words they failed to recall. SRT-total is the total number of recalled words for all trials and has a maximum score of 72. SRT-delayed recall refers to the number of correctly recalled words after a 15-minute delay. SRT-delayed recognition refers to the number of correctly recognized words when each of the 12 words is presented with three distractors.

#### Speed/attention

Speed/attention was defined as the composite score comprising the Wechsler Adult Intelligence Scale-Revised (WAIS-R; [23]) Digit Symbol subtest, the Trail Making Test [24] and the Stroop test. The Digit Symbol test involves writing the symbol corresponding to each single-digit in a list of numbers using a key at the top of the test form as quickly as possible. The time to complete the Trails A (numbers only) from the Trail Making Test was used. Time taken to complete the Stroop Color test, where subjects must name the color of ink used to spell an incongruent words (e.g. the word “blue” written in red ink) as quickly as possible, was also used.

#### Fluid ability

Fluid ability was defined as the composite score comprising the WAIS-III [25]. Letter Number Sequencing subtest and the Matrix Reasoning Test [26] and the Block Design subtest of the WAIS-III. Fluid ability generally refers to novel problem solving and tests of abstract reasoning and the Raven’s matrix reasoning tests tend to have the highest loadings on this construct. A number of studies have found that fluid ability has strong relationships to WCST [27] and to working memory, including the letter number sequencing [27], [28]. The Letter Number Sequencing test involves participants repeating verbally presented lists of intermixed letters and numbers in alphabetical and numerical order. The list lengths increase with each subsequent trial. The Matrix Reasoning subtest requires participants to determine which pattern in a set of eight possible patterns best completes a missing cell of a matrix. The Block Design task gives subjects a score based on their time to complete each item in a series of increasingly complex geometrical shapes; they must replicate each shape seen in a booklet using 4 or 9 identical blocks that are colored half-red and half-white on either side of their diagonals. This is a measure of subjects’ visuospatial manipulation abilities.

#### Lifetime exposure

Lifetime exposure was defined as the composite score comprising years of education and scores on two IQ indices: the NART [29] and WAIS-R vocabulary score [23]. Previous work from our laboratory has demonstrated the validity of this construct using these cognitive tests [20].

### Image Acquisition Procedure

MRI images were acquired in a 3.0T Philips Achieva Magnet using a standard quadrature head coil. A T1-weighted scout image was acquired to determine subject position. One hundred sixty five contiguous 1 mm coronal T1-weighted images of the whole brain were acquired for each subject with an MPRAGE sequence using the following parameters: TR 6.5 ms, TE 3 ms; flip angle 8°, acquisition matrix 256×256 and 240 mm field of view. A neuroradiologist reviewed anatomical scans and any with potentially clinically significant findings, such as abnormal neural structure were removed from the sample prior to the current analysis.

### Freesurfer Methods

Each subject’s structural T1 scans were reconstructed using FreeSurfer [30] (http://surfer.nmr.mgh.harvard.edu/). The accuracy of FreeSurfer’s subcortical segmentation and cortical parcellation [31], [32] has been reported to be comparable to manual labeling. Each subject’s white and gray matter boundaries as well as gray matter and cerebrospinal fluid boundaries were visually inspected slice by slice by an experienced user (DOS), manual control points were added in the case of any visible discrepancy, and reconstruction was repeated until we reached satisfactory results within every subject. The subcortical structure borders were plotted by Freeview visualization tools and compared against the actual brain regions. In case of discrepancy, they were corrected manually. The regions of interest used in this analysis are listed in Table S1 in File S1.

#### Statistical analysis

**Figure 1:**
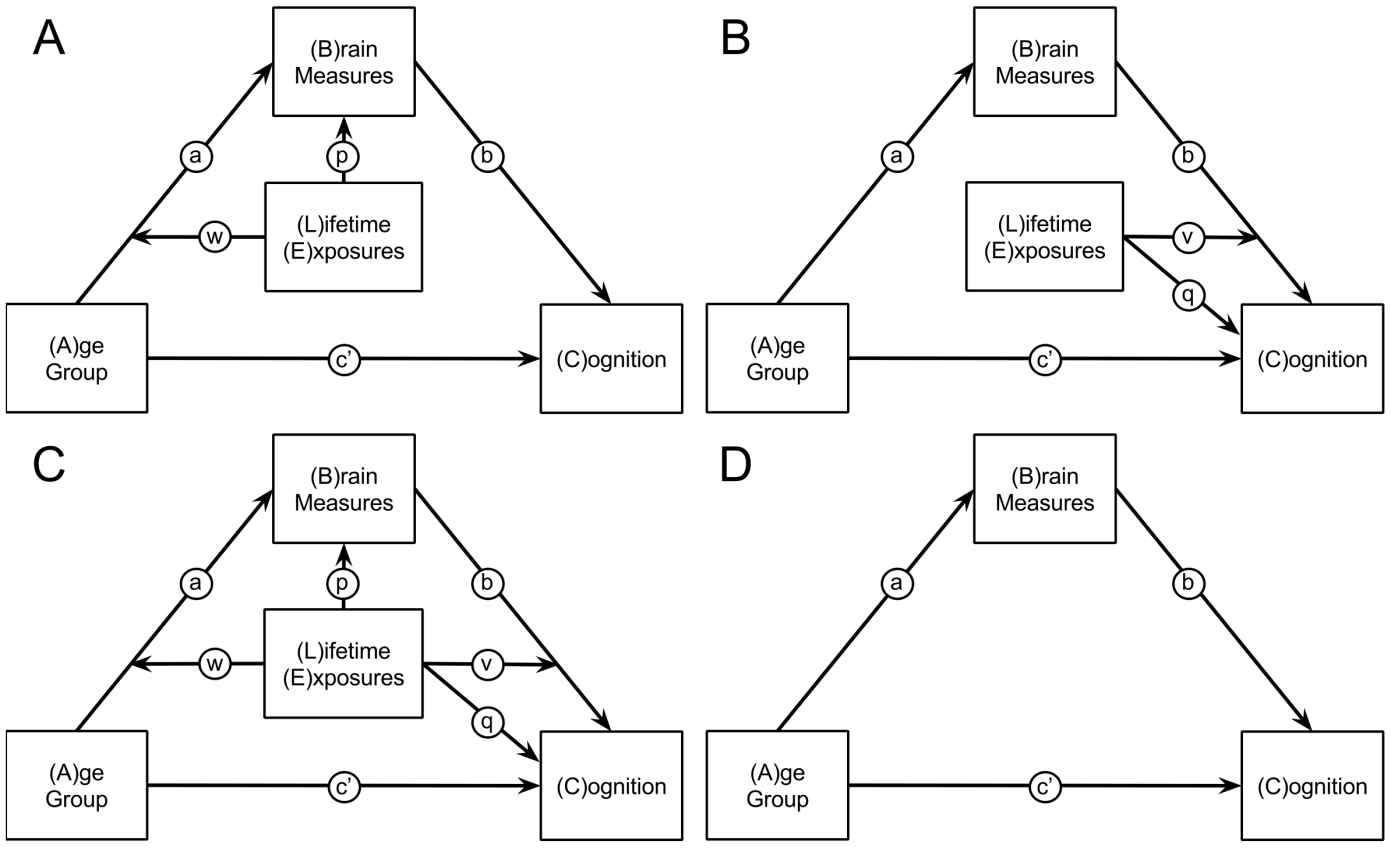
A–D: Structural models testing for the role of lifetime exposures (LE). The letters labeling each arrow in the figure correspond to the parameters in the equations and column headings in the tables of results. The models testing whether LE: A) decreased the effect of advancing age on neural measures, B) decreased the effect of age-related declines in neural measures on cognitive abilities, C) had both of these effects or D) had no effect.

**Figure 2:**
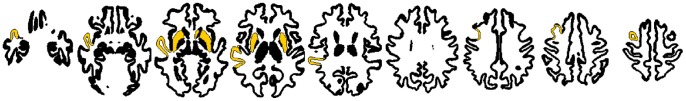
Fluid Ability. Locations where the mediating effect of age group on fluid ability via gray matter volume/thickness is significantly moderated by lifetime exposures. The black underlay is the Freesurfer parcellation of all 84 cortical and subcortical brain regions tested in these analyses. If a brain parcellation is significant at *p(FDR)* <0.05 it is colored yellow.

**Figure 3:**
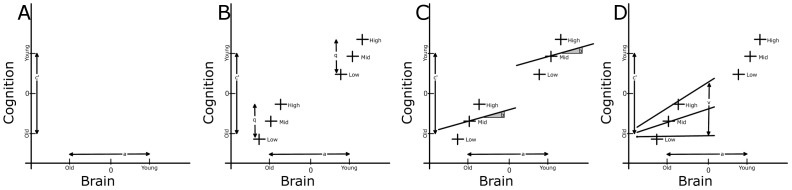
Qualitative illustration of the relationships between brain and cognitive measures for the two age groups at different levels of lifetime exposures. Lifetime exposures was divided into tertiles and referred to as low, medium and high LE. The lines representing the brain-cognition relationships have length equal to the range of values for each tertile of LE. The size of cross hairs on each line is centered at the mean values and has line lengths equal to the standard error. A significant moderation of brain on cognition by LE is evident by a changing slope in the lines as the level of LE changes.

**Figure 4:**
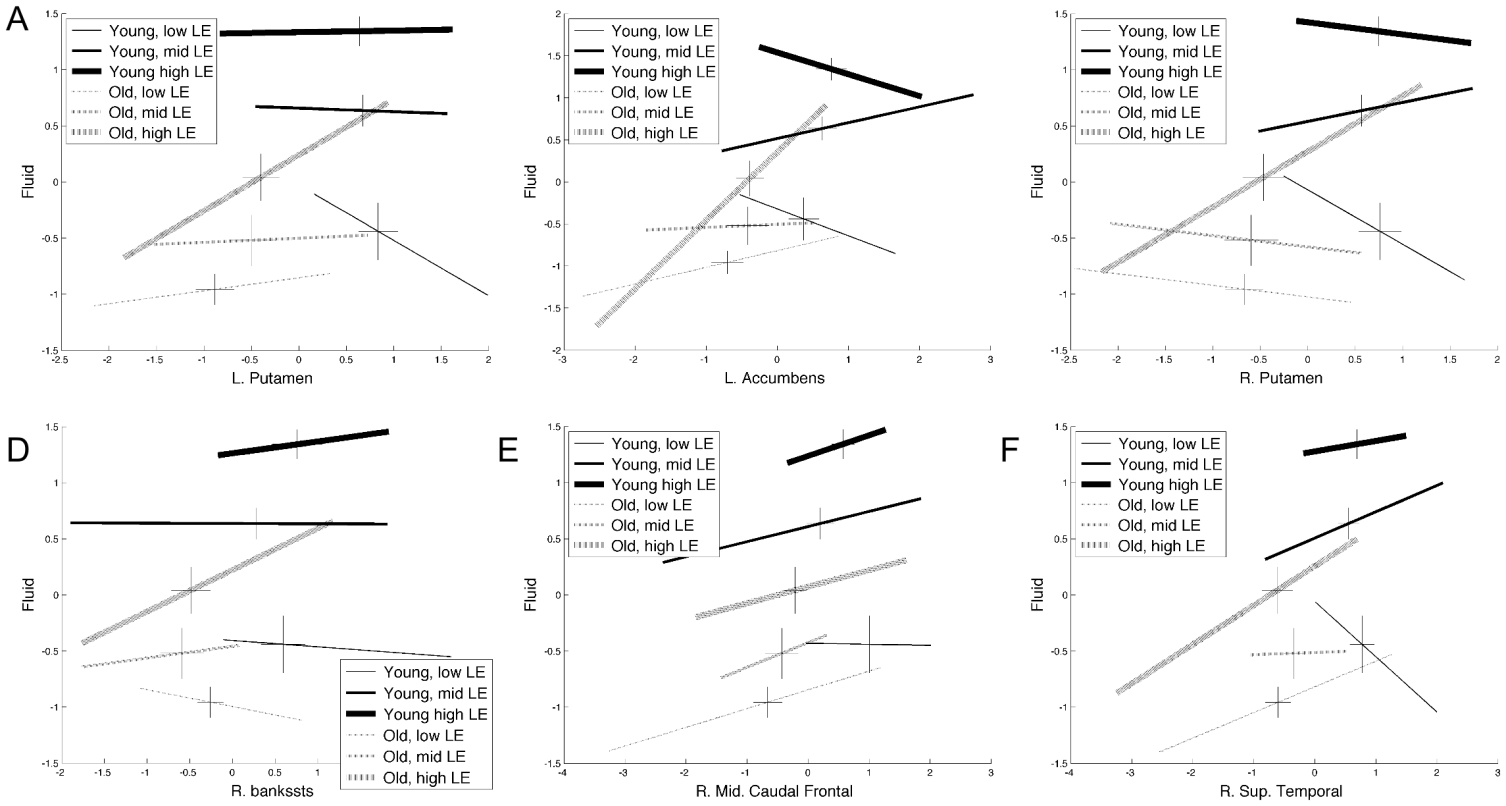
Diagram explaining results by comparing brain measures to cognitive measures for both age groups and various levels of lifetime exposures (LE). This diagram uses results from Model B and the left accumbens volume from Figure 3B as a guide. Parameters refer to those from equations and models in Figure 1. A) Parameter *a* represents a difference between age groups for the brain measure. Parameter c’ represents a difference between age groups for the cognitive measure. B) The crosses plot the brain and cognitive measures against each other for three levels of LE for both age groups. The parameter *q* represents the differing relationship between the brain and cognitive measures for the three different LE groups, low middle and high. C) The parameter *b* represents the overall relationship between the brain and cognitive measures across all levels of LE, i.e. the slope. D) The parameter *v* represents the differing relationship between the brain and cognitive measures for the three LE groups.

To explore how age related neural differences affected cognition and the role of lifetime exposures a statistical path model tested each of the four hypotheses of this study. These were whether LE: 1) decreased the effect of advancing age on neural measures, Figure 1A; 2) decreased the effect of age-related declines in neural measures on cognitive abilities, Figure 1B; 3) had both of these effects, Figure 1C or 4) had no effect, Figure 1D.

The four statistical models tested each of the 84 structural measures derived from the regions of interest extracted from the Freesurfer processing and each of the three cognitive domains. These models were estimated using the regression equations listed below [33]–[35]. Reference to these models uses the associated letters in Figure 1: A, B, C and D. The parameters in the equations correspond to their respective paths in the models, Figure 1. In each model, brain measures of thickness were corrected for mean cortical thickness and brain measures of volume were corrected for normalized brain volume [36]. All models and brain regions were corrected for sex.

1. *B* = *β*_0_+*a*⋅*A*+*p*⋅*LE*+*w*⋅*LE*⋅*A*+*ε*

a. *B* = *β*_0_+(*a*+*w*⋅*LE*)⋅*A*+*p*⋅*LE*+*ε*

2. *C* = *β*_0_+*c*′⋅*A*+*b*⋅*B*+*q*⋅*LE*+*v*⋅*LE*⋅*B*+*ε*

a. *C* = *β*_0_+*c*′⋅*A+*(*b*+*v*⋅*LE*)⋅*B+q*⋅*LE+ε*

3. *B* = *β*_0_+a⋅A+*ε*

4. *C* = *β*_0_+ *c*′⋅*A*+*b*⋅*B*+*ε*

Indirect Effects.

5. Model A.

a. (*a*+*w*⋅*LE*)⋅*b*

b. *a*⋅*b*+*w*⋅*b*⋅*LE*

6. Model B.

a. *a*⋅(*b*+*v*⋅*LE*)

b. *a*⋅*b*+*a*⋅*v*⋅*LE*

7. Model C.

a. (*a*+*w*⋅*LE*)⋅(*b*+*v*⋅*LE*)

b. *a*⋅*b*+(*a*⋅*v+w*⋅*b*)⋅*LE*+*w*⋅*v*⋅*LE* ^2^

8. Model D.

a. *a*⋅*b*

Analyses began by fitting the most complex model; model C, using equations 1 and 2. The regression parameter estimates were then combined to calculate the indirect effect, equation 7. This indirect effect is a function of LE values representing the moderating effect of LE. This equation was tested by probing LE values at the percentiles of: 10, 25, 33, 50, 66, 75 and 90 [37]. Determination of significance for these moderated-mediation effects used bootstrap resampling and confidence intervals at each percentile value of LE. Twenty thousand stratified (by age group) bootstrap resamples were used to determine the bias-corrected percentile confidence intervals [38]–[40]. Six hundred and ninety confidence intervals (CI) were calculated from the bootstrap sampling distributions. The CI ranged from 0.0001 to 0.05 in steps of 0.0001 and from 0.05 to 1 in steps of 0.005. Each CI was tested to determine if it included zero to find the probability level for each brain region. In this way, the approximate *p*-value was calculated (within a small margin of error). Determination of a significant moderated-mediation effect required a two-step procedure. First, multiple comparison correction for the 84 brain measures used the false discovery rate (FDR) of 5 percent [41], [42]. Secondly, only brain regions where the moderator LE interacted with both arms of the path model, parameters *v* and *w* in Figure 1C and equations 1 and 2 were considered [43]. For this screening test a liberal uncorrected threshold of *p*<0.05 was used.

In the absence of both interaction terms being significant in model C, the reduced models A and B were tested. Model A used equations 1 and 4, while model B used equations 2 and 3. The indirect effects for these models are shown in equations 5 and 6, respectively. Testing the indirect effect and the significance of the moderated-mediation effects proceeded in the same manner as for model C, described above. When both interaction terms were non-significant, model D for simple mediation was tested. This model used equations 3 and 4 and the indirect effect is in equation 8. A brain region with a significant mediation effect was determined using only the FDR corrected approach.

All analyses used the publically available and modifiable “Process Models for Neuroimaging” toolbox (https://github.com/steffejr/ProcessModelsNeuroImage) developed by the author JS. This toolbox implements the methods of Preacher and Hayes for use with neuroimaging data. Age group is a categorical variable and the stratified bootstrapping procedure preserved sample sizes in each age group avoiding bias in the resamples due to the different sample sizes in the age groups.

## Results

Several data points were missing from the data set used for analysis due to time limitations in the administration of the tasks, as well as several instances of administrator error. In several cases the neuropsychological battery was cut short to accommodate the schedule of the MRI scanning center. These abbreviated batteries were unrelated to participant speed, but reflected limitations due to the duration of the battery. The most affected measures included the WAIS-III tasks: Vocab, Matrix Reasoning, and Blocks. Administration errors resulted in missing values in Stroop, Trail-Making Test, and SRT and did not reflect participant performance.

Speed: Speed variables derived from the Stroop color word task were missing for a total of three participants: two old and one young participant. The Digit-Symbol and Trail-Making Test variables were fully intact. Fluid ability: Scores on the WAIS-III Matrix Reasoning task were missing from three old and three young participants. Scores on the WAIS-III Blocks task were missing from five old and one young participant. The Letter-Number Sequencing task was intact for all participants. Memory: For variables derived from the SRT, one young participant was missing the task completely, while two other young and two old participants were missing two variables each (SRT delayed recall and SRT delayed recognition). One additional older participant was also missing the delayed recognition variable. Lifetime exposures: Scores on the WAIS-III Vocabulary task were missing from one young and five old participants.

With regards to the mediation models, there were significant results for only model B for fluid ability. Figure 2 shows all regions of interest tested in this analysis and those regions having significant moderated-mediation effects in yellow. Parameter estimates for the significant regions are in Table 2 along with the indirect effect sizes from probing various levels of LE. An overall observation is that these results support the theory of cognitive reserve, and inclusion of LE into the model identified brain-cognition relationships that would have been missed in its absence. The idea of uncovering brain-cognitive relationships is supported by the lack of any significant findings from the simpler mediation model when testing fluid ability.

**Table 2:** Fluid ability, Model B.

								Percentiles						
Region	Hemi	Measure	a	c’	b	q	v	10	25	34	50	66	75	90
Putamen	L	V	−1.2**	−1.0**	0.1	0.7**	0.2*	0.2	0.1	0	−0.1	−0.3	−0.4	−0.4**
Accumbens	L	V	−1.0**	−1.0**	0.2*	0.7**	0.3**	0.2	0	−0.1	−0.2	−0.4**	−0.4**	−0.5**
Putamen	R	V	−1.1**	−1.1**	0.1	0.7**	0.3**	0.3	0.2	0.1	−0.1	−0.3	−0.3**	−0.4**
Bank of the superior temporal sulcus	R	T	−0.8**	−1.0**	0.1	0.7**	0.2*	0.1	0.1	0	−0.1	−0.1	−0.2**	−0.2**
Middle Caudal Frontal	R	T	−0.9**	−1.1**	0.1	0.7**	0.2*	0.1	0.1	0	−0.1	−0.2	−0.2**	−0.3**
Superior Temporal	R	T	−1.1**	−0.9**	0.2	0.7**	0.2*	0.1	0	−0.1	−0.2	−0.3	−0.4**	−0.4**

Notes: Hemi: hemisphere, Measure: (T)hickness or (V)olume, a: parameter relating age group to brain measure, c’: parameter relating age group to cognitive measure, b: parameter relating brain measure to cognitive measure, q: parameter relating lifetime exposure (LE) to cognitive measure, v: interaction of brain measure and LE in predicting cognitive measure. The last seven columns are the indirect effects of age group on the cognitive measure via the brain measure probed at percentiles of LE.

**significant at p(FDR) <0.05;

*significant at p(uncorrected) <0.05.

The mediating effect of advancing age on fluid ability via the volume of three subcortical regions and the mean thickness of four cortical regions was moderated by LE. These regions included bilateral putamen volume, left accumbens, the bank of the right superior sulcus, right middle frontal gyrus, right posterior central gyrus and the right superior temporal gyrus mean thickness. No results from the other models nor from any of the models for speed and memory were significant after correcting for multiple comparisons.

Using FDR correction showed six brain regions having significant moderated-mediation effects on fluid ability. If uncorrected p-values were used 16 brain measures were identified. For the models that had zero FDR corrected significant results, there were the following number of regions with significant uncorrected results: Model A, fluid ability: 2, memory: 1, speed: 1; Model B, memory: 5, speed: 1; Model C, fluid ability: 1, memory: 0, speed: 0; Model D, fluid ability: 10, memory: 5, speed: 8. These uncorrected results are presented in Tables S2–S13 in File S1.

### Exploration of Significant Moderation Models

To aid understanding of the moderated-mediation results, Figure 3 presents line plots of each of the significant brain regions. Brain measure and cognitive performances are represented on the x and y-axes respectively. Lifetime exposure scores were divided into tertiles (low, medium and high) and relationships between brain measures and cognition are graphed for each of these LE tertiles in young and old subjects. The lines representing the brain-cognition relationships have length equal to the range of values for the brain measure for each of the 6 groups (young at each of the three levels of LE and old also at these three levels of LE). The cross hairs on each line are centered at the standardized mean values of the brain measure and cognition for each group and have lengths equal to the respective standard errors of the two variables. This representation of the data facilitates exploration of the mediation and moderation effects.

To aid in the interpretation of the results from model B presented in Figure 3 and Table 2, a diagram is presented in Figure 4 roughly based off the findings in the left Accumbens, Figure 3B. In Figure 4A the effect of age group on the brain and cognitive measures is represented by parameters *a* and *c’* respectively, which are from equations 3 and 2, respectively. Panel B plots the relationship between the brain and cognitive measures for the three tertiles of LE and the two age groups. The increasing cognitive values for increasing LE are captured by parameter *q* in equation 2. Although not included in this diagram, the increasing brain measures with increasing LE would be represented by parameter *p* from equation 1. Parameter *b* in equation 2 and panel C captures the overall relationship between the brain and cognitive measures. The interaction term in equation 2 is parameter *v* in panel D and demonstrates that the relationship between the brain and cognitive measures increase with increasing LE.

General interpretations of these results follow based on inspection of Table 2 and Figure 3 using Figure 4 as a guide. Lifetime exposures did not alter the effect of advancing age on the brain measures. This is demonstrated in Figure 3, because the mean brain measures within each tertile are not significantly different from each other. This is seen by the overlapping standard errors in the horizontal cross hairs for each LE tertials. Lifetime exposures had a greater effect on the fluid ability measures as evidenced by the large spread of fluid ability values for the three tertiles and the highly significant *q* parameters. Significant moderated-mediation effects occurred at the higher levels of LE. This effect was largely driven by the old age group as evidenced by the relatively steep slopes of the brain-cognitive relationship at the highest LE tertile.

These findings demonstrate that those with LE values in the 66th, 75th and 90th percentiles are better able to deal with age-related differences in brain measures. These results are more interpretable in their original units. Within the older age group these percentiles correspond to mean years of education of 18.8, 19.3, 20; NART errors of 3.3, 3.5, 3; and WAIS vocabulary of 63.0, 63.1, 63.8.

The models tested assume a causal relationship between the variables; however, significant test statistics do not prove causality they only support the theory. Further support for the hypothetical models is the finding of non-significant results from alternate models with the same data [44]. Alternate models are ones where the variables in the model are switched and the models are re-tested. This may result in physiologically and theoretically implausible models; however, if the data support an alternate (non-plausible) model then support for the hypothesized model is diminished. Not all possible alternate models for all brain regions were tested; however, findings for the left accumbens were. The left accumbens volume supported model B, where LE moderated the effect of brain volume on fluid ability. With LE moderating the path between brain volume and fluid ability the causal pathway was reversed and when the causal pathway went from age group to fluid ability to brain volume, the indirect effects and interactions were not significant. Although far from exhaustive, these findings support the hypothesized models in this study.

## Discussion

This work explored the role of lifetime exposures (LE) to education and verbal abilities in moderating the effects of age-related differences in gray matter measures on cognition using statistical process models. The results from this study support the role of LE as a proxy for cognitive reserve (CR) and not brain maintenance. The findings in all brain regions demonstrate a stronger positive relationship between the brain measures and fluid ability in older adults with larger LE measures. Even though there was an increase in the strength of the relationships between brain and fluid ability in older adults with larger LE measures, the range of brain measure values did not increase. Therefore, increased LE did not increase the range of brain measures. This is shown in Figure 3 by the length of the lines and further supported by the lack of significant findings supporting the brain maintenance model. An increase in the strength of this relationship means that those with greater LE have better fluid ability even at the same value of the brain measures. Additionally, the level of LE did not differ between the age groups. This suggests that those older adults with greater LE are able to take better advantage of the brain structure they have than their counterparts with lower LE values.

The finding of stronger neural-cognitive relationships with greater LE supports the idea that the mechanistic implementation of CR is neural reserve [6]. Neural reserve refers to the modifiable individual differences in cognitive processing, such as efficiency and capacity of a brain region (see [6] for a review). Efficiency is the rate at which brain activity increases to meet increasing cognitive demands. Capacity is the cognitive load at which the maximum amount of brain activity is reached. Neural reserve therefore describes relationships between neural function and cognition occurring in all individuals and is not disease specific. An individual with high CR would therefore have greater neural efficiency and capacity [45] leading to stronger neural-cognitive relationships.

This interpretation of LE as facilitating better performance given the same amount of brain volume complements previous observations in Alzheimer’s disease supporting the theory of CR. Earlier work showed that when controlling for clinical severity, patients with higher LE had more advanced AD pathology. This first such observation was a report of an inverse relationship between regional cerebral blood flow and education in Alzheimer’s disease patients matched for clinical severity [46]. In this study cerebral blood flow was used as a proxy for AD pathology. Subsequent reports demonstrated comparable results using direct measures of pathology at autopsy, or using newer markers for pathology such as amyloid PET or CSF Aβ. These observations suggest that patients with higher LE can tolerate more pathology because their remaining neural tissue is either more efficient, has greater capacity or can compensate more effectively. This allows them to remain clinically equivalent to patients with lower LE who have less severe pathology. The present analyses differ in that we did not match subjects for cognitive performance; rather we explored the relationship between neural measures and cognitive performance as moderated by LE. Thus, we report that given a specific quantity of gray matter within some brain regions, individuals with higher LE perform better. The key observation is that in both sets of analyses, LE is associated with more effective utilization of neural resources.

Lifetime exposures moderated the effect that age-related differences in gray matter has on fluid ability within the subcortical volumes of the bilateral putamen, left accumbens and the cortical thickness of the left bank of the superior temporal sulcus, middle caudal frontal gyrus, right postcentral gyrus and the superior temporal gyrus. Previous developmental work showed that the volume of the putamen is related to intelligence in children [47]. The current results suggest that the combination of both the volume of the putamen and intelligence has a greater influence on fluid abilities then either alone. Previous work also showed that while advancing age increased the variability of brain activity, the variability of brain activity within the accumbens mediated the effect of age on risk-taking ability [48]. Although not tested in our work or theirs, the union of these findings raises the question of whether the age-related structural and functional effects, which are both related to cognitive measures, are themselves related or represent independent effects. Previous work demonstrated an age-related difference in the volume of superior temporal sulcus that was related to cognitive differences in rhesus monkeys and therefore not related to any Alzheimer’s pathology [49]. Using this as a speculative analogy, it supports the idea that our results are from normal aging and not preclinical disease related pathology.

The current results found no evidence to support the brain maintenance (BM) hypothesis. This could have occurred for a number of reasons. One is that the measures of LE used could simply be better proxies of CR than BM; therefore, biasing the results towards support of CR. Another is that support for BM would come from a moderating effect of age group on brain measures. The values calculated using Freesurfer might be considered accurate measures of brain volume and thickness. Similarly, the composite cognitive measures may accurately capture cognitive abilities. However, age group is a crude measure of advancing age. Therefore, support for the BM hypothesis requires the interaction between a crude and accurate measurement, while support for CR results from the interaction between two accurate measures. It is also possible that the data itself lends itself better to the CR model than the BM model. This idea is based off the large difference in the coefficient of variability between the brain measures (0.36 for old and 0.21 for young in the volume of the left Accumbens) and the cognitive measures (1.82 for old and 1.64 for young in the fluid ability measure). Therefore, there is more variance in the cognitive measure to capture by including an interacting term than in the brain measure. Another possibility is that age group captures a large portion of the brain measures leaving little residual variance for LE to account for. In the same line of thought, age group and the brain measures may leave a large amount of residual variance in the cognitive measures facilitating the ability to find a relationship with LE. Within the left accumbens again, age group, sex, and normalized whole brain volume (nWBV) accounted for 36% of the variance. While an equal amount of the variance of fluid ability was accounted for by age group, sex, nWBV and left accumbens volume. Therefore, for at least this brain region both regression models left equal amounts of residual variance. Inclusion of LE and its interaction with left accumbens volume accounted for an additional 31% of the variance in fluid ability.

The effect of advancing age on structural brain measures only explained age-related differences in fluid ability and this relationship was dependent on the individual levels of LE. Using statistical models that did not include LE measures would not have identified these brain regions as having an influence on fluid ability. Some aspects of the current work are similar to previous work from our group and it is important to compare the two [50]. The current work used a univariate approach and tested each brain region identified with the Freesurfer software independently, while previous work identified covariance brain patterns related to cognitive domains and then demonstrated that the brain covariance patterns accounted for nearly all of the age related variance in the cognitive scores. The current work builds off this finding that measures of gray matter are affected by advancing age and account for age related variance in cognition. Another difference is that the current work uses models with implied causality while previous work identified relationships and their relative strengths, without any causal assumptions.

The statistical models used in this work are referred to as “process” models in the statistical literature and are relatively novel to the neuroimaging community; however, they are well established in the communications literature. The important distinction of process models is their inclusion of moderating, or interaction, effects, which are not included in mediation analyses. In this way mediation analyses, which are not new to neuroimaging [9], represent a specific statistically degenerate case of process models. The implementation and testing of these models represents an approach for testing our previously described conceptual research model of the neural basis of CR [51]. Although the current work represents a small piece of the more comprehensive research model by focusing only on age-related structural differences, it lays the groundwork for further explorations into how these structural effects interact with measures of functional activity. It is plausible to assume that functional networks involved in cognitive abilities rely on the brain structures identified here and CR increases their functional abilities or efficiency. This is speculation; however, it is possible to test for the role that aging has on the gray matter integrity underlying functional activation [52].

The LE measures chosen for this study have previously been used as CR proxies. Future directions will explore other measures of LE. It is plausible that both theories of BM and CR are at work throughout our lifetime and are supported in different brain regions by different lifetime exposure measures. A limitation of this study is the relatively small sample size. Correction for multiple comparisons highlighted the most-significant findings and uncorrected results are included as supplementary material. The expanse of uncorrected results suggests that future work with larger samples may shed greater insight into the role of LE. For example, future models could include tests of third order interactions between age, brain measures and LE. One caution with the current results is that causal models with cross sectional data do not prove causal relationships; they demonstrate support for causation in a model. In the current work, the assumption was made that the differences in gray matter measurements resulted from advancing age as demonstrated by published longitudinal studies [2]. The assumption was also made that age-related differences in cognitive abilities were partially due to age-related differences in cortical measurements. We feel that the assumed causal pathways are justified, but recognize that unmeasured effects may also play a role in these relationships. Future work with an expanded set of brain measures will explore this.

## Conclusions

The concepts of brain maintenance and cognitive reserve suggest that individual differences in advantageous lifetime exposures affect the relationships between advancing age and neural measures and between neural measures and cognitive outcomes, respectively. The current work suggests that the role of these mechanisms differ throughout the brain and across different cognitive domains. Understanding the mechanistic role of such protective and compensatory factors has important implications for interventional strategies. The main findings of this work demonstrates that the impact of differences in gray matter volume and thickness on cognition is moderated by LE, consistent with the predictions of the cognitive reserve model, and thus is potentially modifiable by supplying appropriate experiences. Therefore, intervention strategies to preserve cognitive abilities in the face of advancing age may train individuals to better utilize the brain matter that they currently have, even in the face of age-related decreasing volumes and thicknesses. Better understanding of these effects requires further research to investigate subtle cognitive reserve effects and to identify the most feasible cognitive interventions that have the greatest positive effect on cognitive performance.

## Supporting Information

File S1 **This file includes a series of tables (Table S1–Table S13) with additional information that may be of interest to the reader.** There is a table containing all Freesurfer derived regions of interest with their group mean values used in these analyses. Additionally, results are presented when an uncorrected height threshold of *p*<0.05 is used. This demonstrates extensive support for the cognitive reserve and brain maintenance theories that did not exceed the stringent thresholds used. Table S1, Freesurfer derived measurements used and group means and standard deviations. Table S2, Memory, Model A. Table S3, Memory, Model B. Table S4, Memory, Model C. Table S5, Memory, Model D. Table S6, Speed, Model A. Table S7, Speed, Model B. Table S8, Speed, Model C. Table S9, Speed, Model D. Table S10, Fluid Ability, Model A. Table S11, Fluid Ability, Model B. Table S12, Fluid Ability, Model C. Table S13, Fluid Ability, Model D.
(DOCX)
