# Supplementary material for: The Role of Education and Verbal Abilities in Altering the Effect of Age-Related Gray Matter Differences on Cognition
Source: PLoS One. 2014 Mar 13;9(3):e91196. doi: 10.1371/journal.pone.0091196 (PMC3953380; doi:10.1371/journal.pone.0091196)
Supplement: File S1 — This file includes a series of tables (Table S1–Table S13) with additional information that may be of interest to the reader. There is a table containing all Freesurfer derived regions of interest with their group mean values used in these analyses. Additionally, results are presented when an uncorrected height threshold of p<0.05 is used. This demonstrates extensive support for the cognitive reserve and brain maintenance theories that did not exceed the stringent thresholds used. Table S1, Freesurfer derived measurements used and group means and standard deviations. Table S2, Memory, Model A. Table S3, Memory, Model B. Table S4, Memory, Model C. Table S5, Memory, Model D. Table S6, Speed, Model A. Table S7, Speed, Model B. Table S8, Speed, Model C. Table S9, Speed, Model D. Table S10, Fluid Ability, Model A. Table S11, Fluid Ability, Model B. Table S12, Fluid Ability, Model C. Table S13, Fluid Ability, Model D. (DOCX) [file pone.0091196.s001.docx]

| Table S1. Freesurfer derived measurements used and group means and standard deviations. | | | | | | |
| --- | --- | --- | --- | --- | --- | --- |
| Region |  | Hemi | Measure Type | Young mean (std) |  | Old mean (std) |
| Thalamus |  | L | V | 7200 (970) |  | 6700 (1070) |
| Caudate |  | L | V | 3790 (562) |  | 3240 (480) |
| Putamen |  | L | V | 5760 (622) |  | 4500 (780) |
| Pallidum |  | L | V | 1700 (253) |  | 1570 (487) |
| Hippocampus |  | L | V | 4310 (398) |  | 3780 (444) |
| Amygdala |  | L | V | 1680 (171) |  | 1400 (282) |
| Accumbens |  | L | V | 660 (142) |  | 450 (164) |
| VentralDC |  | L | V | 3900 (598) |  | 3440 (520) |
| Thalamus |  | R | V | 7000 (740) |  | 6400 (780) |
| Caudate |  | R | V | 3860 (550) |  | 3270 (521) |
| Putamen |  | R | V | 5500 (574) |  | 4500 (720) |
| Pallidum |  | R | V | 1620 (220) |  | 1370 (355) |
| Hippocampus |  | R | V | 4320 (493) |  | 3920 (452) |
| Amygdala |  | R | V | 1780 (232) |  | 1560 (282) |
| Accumbens |  | R | V | 720 (98) |  | 540 (118) |
| VentralDC |  | R | V | 3900 (630) |  | 3460 (479) |
| Bank of the superior temporal sulcus |  | L | T | 2.7 (0.17) |  | 2.47 (0.204) |
| Anterior Caudal Cingulate |  | L | T | 2.83 (0.255) |  | 2.73 (0.266) |
| Middle Caudal Frontal |  | L | T | 2.72 (0.144) |  | 2.57 (0.157) |
| Cuneus |  | L | T | 1.89 (0.133) |  | 1.75 (0.165) |
| Entorhinal |  | L | T | 3.30 (0.377) |  | 3.15 (0.411) |
| Fusiform |  | L | T | 2.79 (0.142) |  | 2.67 (0.144) |
| Inferior Parietal |  | L | T | 2.75 (0.137) |  | 2.51 (0.149) |
| Inferior Temporal |  | L | T | 2.99 (0.162) |  | 2.88 (0.140) |
| Isthmus of Cingulate |  | L | T | 2.70 (0.209) |  | 2.5 (0.18) |
| Lateral Occipital |  | L | T | 2.32 (0.159) |  | 2.17 (0.141) |
| Lateral Orbitofrontal |  | L | T | 2.74 (0.210) |  | 2.55 (0.156) |
| Lingual |  | L | T | 2.05 (0.103) |  | 1.89 (0.128) |
| Medial Orbitofrontal |  | L | T | 2.5 (0.18) |  | 2.34 (0.201) |
| Middle Temporal |  | L | T | 3.12 (0.149) |  | 2.92 (0.176) |
| Parahippocampal |  | L | T | 2.75 (0.352) |  | 2.60 (0.263) |
| Paracentral |  | L | T | 2.52 (0.134) |  | 2.3 (0.18) |
| Parsopercularis |  | L | T | 2.76 (0.136) |  | 2.54 (0.136) |
| Parsorbitalis |  | L | T | 2.89 (0.250) |  | 2.62 (0.195) |
| Parstriangularis |  | L | T | 2.7 (0.17) |  | 2.43 (0.196) |
| Pericalcarine |  | L | T | 1.60 (0.152) |  | 1.50 (0.157) |
| Posterior Central |  | L | T | 2.19 (0.131) |  | 2.05 (0.160) |
| Posterior Cingulate |  | L | T | 2.72 (0.180) |  | 2.51 (0.146) |
| Precentral |  | L | T | 2.71 (0.103) |  | 2.48 (0.165) |
| Precuneus |  | L | T | 2.51 (0.122) |  | 2.33 (0.160) |
| Rostral Anterior Cingulate |  | L | T | 3.10 (0.216) |  | 2.86 (0.216) |
| Rostral Middle Frontal |  | L | T | 2.53 (0.118) |  | 2.39 (0.146) |
| Superior Frontal |  | L | T | 2.95 (0.155) |  | 2.69 (0.157) |
| Superior Parietal |  | L | T | 2.37 (0.130) |  | 2.2 (0.17) |
| Superior Temporal |  | L | T | 2.96 (0.157) |  | 2.74 (0.161) |
| Supramarginal |  | L | T | 2.76 (0.133) |  | 2.54 (0.161) |
| Frontal Pole |  | L | T | 2.93 (0.381) |  | 2.73 (0.361) |
| Temporal Pole |  | L | T | 3.60 (0.453) |  | 3.32 (0.508) |
| Trans-Temporal |  | L | T | 2.51 (0.216) |  | 2.22 (0.215) |
| Insula |  | L | T | 3.22 (0.141) |  | 2.98 (0.183) |
| Bank of the superior temporal sulcus |  | R | T | 2.67 (0.219) |  | 2.47 (0.176) |
| Anterior Caudal Cingulate |  | R | T | 2.81 (0.228) |  | 2.64 (0.268) |
| Middle Caudal Frontal |  | R | T | 2.7 (0.17) |  | 2.47 (0.171) |
| Cuneus |  | R | T | 1.94 (0.129) |  | 1.75 (0.109) |
| Entorhinal |  | R | T | 3.35 (0.450) |  | 3.25 (0.351) |
| Fusiform |  | R | T | 2.75 (0.173) |  | 2.6 (0.17) |
| Inferior Parietal |  | R | T | 2.61 (0.137) |  | 2.46 (0.126) |
| Inferior Temporal |  | R | T | 2.94 (0.156) |  | 2.8 (0.20) |
| Isthmus of Cingulate |  | R | T | 2.64 (0.212) |  | 2.42 (0.216) |
| Lateral Occipital |  | R | T | 2.33 (0.151) |  | 2.21 (0.165) |
| Lateral Orbitofrontal |  | R | T | 2.74 (0.163) |  | 2.48 (0.195) |
| Lingual |  | R | T | 2.10 (0.124) |  | 1.92 (0.126) |
| Medial Orbitofrontal |  | R | T | 2.66 (0.213) |  | 2.43 (0.201) |
| Middle Temporal |  | R | T | 3.09 (0.146) |  | 2.83 (0.194) |
| Parahippocampal |  | R | T | 2.69 (0.361) |  | 2.52 (0.264) |
| Paracentral |  | R | T | 2.50 (0.109) |  | 2.30 (0.199) |
| Parsopercularis |  | R | T | 2.77 (0.181) |  | 2.48 (0.198) |
| Parsorbitalis |  | R | T | 2.87 (0.256) |  | 2.56 (0.229) |
| Parstriangularis |  | R | T | 2.66 (0.140) |  | 2.38 (0.182) |
| Pericalcarine |  | R | T | 1.66 (0.145) |  | 1.55 (0.143) |
| Posterior Central |  | R | T | 2.16 (0.119) |  | 2.00 (0.189) |
| Posterior Cingulate |  | R | T | 2.67 (0.144) |  | 2.45 (0.153) |
| Precentral |  | R | T | 2.66 (0.151) |  | 2.42 (0.191) |
| Precuneus |  | R | T | 2.45 (0.125) |  | 2.30 (0.135) |
| Rostral Anterior Cingulate |  | R | T | 3.13 (0.229) |  | 2.87 (0.244) |
| Rostral Middle Frontal |  | R | T | 2.55 (0.154) |  | 2.4 (0.17) |
| Superior Frontal |  | R | T | 2.93 (0.153) |  | 2.66 (0.184) |
| Superior Parietal |  | R | T | 2.29 (0.104) |  | 2.19 (0.139) |
| Superior Temporal |  | R | T | 2.9 (0.16) |  | 2.64 (0.210) |
| Supramarginal |  | R | T | 2.69 (0.137) |  | 2.47 (0.147) |
| Frontal Pole |  | R | T | 2.94 (0.381) |  | 2.71 (0.325) |
| Temporal Pole |  | R | T | 3.7 (0.37) |  | 3.4 (0.39) |
| Trans-Temporal |  | R | T | 2.54 (0.230) |  | 2.29 (0.289) |
| Insula |  | R | T | 3.1 (0.19) |  | 2.95 (0.198) |
| Notes. L and R refer to left and right hemispheres respectively. V and T refer to volume of grey matter in a region and average thickness of a cortical region, respectively. | | | | | | |

| Table S2. Memory, Model A | | | | | | | | | | | | | | | |
| --- | --- | --- | --- | --- | --- | --- | --- | --- | --- | --- | --- | --- | --- | --- | --- |
|  |  |  |  |  |  |  |  |  | Percentiles | | | | | | |
| Region | Hemi | Measure | a | p | w | c' | b |  | 10 | 25 | 34 | 50 | 66 | 75 | 90 |
| Parsorbitalis | L | T | -1.0* | -0.6* | 0.7* | -1.2* | -0.2 |  | 0.340* | 0.290* | 0.247* | 0.169* | 0.107* | 0.076 | 0.048 |
| Notes: Hemi: hemisphere, Measure: (T)hickness or (V)olume, a: parameter relating age group to brain measure, p: parameter relating lifetime exposure (LE) to brain measure, w: interaction parameter of age group and LE in predicting brain measure, c’: parameter relating age group to cognitive measure, b: parameter relating brain measure to cognitive measure. The last seven columns are the indirect effects of age group on the cognitive measure via the brain measure probed at percentiles of LE. *significant at *p*(uncorrected) < 0.05. | | | | | | | | | | | | | | | |

| Table S3. Memory, Model B | | | | | | | | | | | | | | | |
| --- | --- | --- | --- | --- | --- | --- | --- | --- | --- | --- | --- | --- | --- | --- | --- |
|  |  |  |  |  |  |  |  |  | Percentiles | | | | | | |
| Region | Hemi | Measure | a | c' | b | q | v |  | 10 | 25 | 34 | 50 | 66 | 75 | 90 |
| Hippocampus | L | V | -1.1* | -1.2* | -0.2 | 0.5* | 0.2* |  | 0.5* | 0.4* | 0.3* | 0.2 | 0 | 0 | -0.1 |
| Putamen | R | V | -1.1* | -1.0* | 0.1 | 0.5* | 0.2* |  | 0.2 | 0.1 | 0 | -0.2 | -0.3 | -0.3* | -0.4* |
| Hippocampus | R | V | -0.7* | -1.1* | 0 | 0.5* | 0.2* |  | 0.2* | 0.2 | 0.1 | 0 | -0.1 | -0.1 | -0.2 |
| Lingual | L | T | -1.0* | -1.1* | 0 | 0.5* | 0.2* |  | 0.3 | 0.2 | 0.1 | -0.1 | -0.2 | -0.2* | -0.3* |
| Lingual | R | T | -1.0* | -1.0* | 0.1 | 0.6* | 0.2* |  | 0.2 | 0.1 | 0 | -0.1 | -0.2 | -0.2 | -0.3* |
| Notes: Hemi: hemisphere, Measure: (T)hickness or (V)olume, a: parameter relating age group to brain measure, c’: parameter relating age group to cognitive measure, b: parameter relating brain measure to cognitive measure, q: parameter relating lifetime exposure (LE) to cognitive measure, v: interaction parameter of LE and brain measure in predicting cognitive measure. The last seven columns are the indirect effects of age group on the cognitive measure via the brain measure probed at percentiles of LE. *significant at *p*(uncorrected) < 0.05. | | | | | | | | | | | | | | | |

| Table S4. Memory, Model C | | | | | | | | | | | | | | | | | |
| --- | --- | --- | --- | --- | --- | --- | --- | --- | --- | --- | --- | --- | --- | --- | --- | --- | --- |
|  |  |  |  |  |  |  |  |  |  |  | Percentiles | | | | | | |
| Region | Hemi | Measure | a | p | w | c' | b | q | v |  | 10 | 25 | 34 | 50 | 66 | 75 | 90 |
| No Results |  |  |  |  |  |  |  |  |  |  |  |  |  |  |  |  |  |
| Notes: Hemi: hemisphere, Measure: (T)hickness or (V)olume, a: parameter relating age group to brain measure, p: parameter relating lifetime exposures (LE) to brain measure, w: interaction parameter of age group and LE in predicting brain measure, c’: parameter relating age group to cognitive measure, b: parameter relating brain measure to cognitive measure, q: parameter relating lifetime exposure (LE) to cognitive measure, v: interaction parameter of LE and brain measure in predicting cognitive measure. The last seven columns are the indirect effects of age group on the cognitive measure via the brain measure probed at percentiles of LE. *significant at *p*(uncorrected) < 0.05. | | | | | | | | | | | | | | | | | |

| Table S5. Memory, Model D | |  |  |  |  |
| --- | --- | --- | --- | --- | --- |
| Region | Hemi | Measure | a | c' | b |
| Accumbens | L | V | -1.0* | -0.8* | 0.2* |
| Accumbens | R | V | -1.2* | -0.6* | 0.3* |
| Parsorbitalis | L | T | -1.0* | -1.2* | -0.2 |
| Trans. Temporal | L | T | -1.0* | -0.8* | 0.3* |
| Insula | L | T | -1.0* | -0.8* | 0.2 |

Notes: Hemi: hemisphere, Measure: (T)hickness or (V)olume, a: parameter relating age group to brain measure, c’: parameter relating age group to cognitive measure, b: parameter relating brain measure to cognitive measure. *significant at *p*(uncorrected) < 0.05.

| Table S6. Speed, Model A | | | | | | | | | | | | | | | |
| --- | --- | --- | --- | --- | --- | --- | --- | --- | --- | --- | --- | --- | --- | --- | --- |
|  |  |  |  |  |  |  |  |  | Percentiles | | | | | | |
| Region | Hemi | Measure | a | p | w | c' | b |  | 10 | 25 | 34 | 50 | 66 | 75 | 90 |
| Isthmus of Cingulate | R | T | -0.8* | -0.1 | 0.5* | -1.0* | 0.2* |  | -0.294* | -0.252* | -0.217 | -0.153* | -0.102* | -0.077 | -0.055 |
| Notes: Hemi: hemisphere, Measure: (T)hickness or (V)olume, a: parameter relating age group to brain measure, p: parameter relating lifetime exposure (LE) to brain measure, w: interaction parameter of age group and LE in predicting brain measure, c’: parameter relating age group to cognitive measure, b: parameter relating brain measure to cognitive measure. The last seven columns are the indirect effects of age group on the cognitive measure via the brain measure probed at percentiles of LE. *significant at *p*(uncorrected) < 0.05. | | | | | | | | | | | | | | | |

| Table S7. Speed, Model B | | | | | | | | | | | | | | | |
| --- | --- | --- | --- | --- | --- | --- | --- | --- | --- | --- | --- | --- | --- | --- | --- |
|  |  |  |  |  |  |  |  |  | Percentiles | | | | | | |
| Region | Hemi | Measure | a | c' | b | q | v |  | 10 | 25 | 34 | 50 | 66 | 75 | 90 |
| Accumbens | L | V | -1.0* | -1.3* | 0.1 | 0.5* | 0.2* |  | 0.1 | 0.1 | 0 | -0.1 | -0.2* | -0.3* | -0.3* |
| Notes: Hemi: hemisphere, Measure: (T)hickness or (V)olume, a: parameter relating age group to brain measure, c’: parameter relating age group to cognitive measure, b: parameter relating brain measure to cognitive measure, q: parameter relating lifetime exposure (LE) to cognitive measure, v: interaction parameter of LE and brain measure in predicting cognitive measure. The last seven columns are the indirect effects of age group on the cognitive measure via the brain measure probed at percentiles of LE. *significant at *p*(uncorrected) < 0.05. | | | | | | | | | | | | | | | |

| Table S8. Speed, Model C | | | | | | | | | | | | | | | | | |
| --- | --- | --- | --- | --- | --- | --- | --- | --- | --- | --- | --- | --- | --- | --- | --- | --- | --- |
|  |  |  |  |  |  |  |  |  |  |  | Percentiles | | | | | | |
| Region | Hemi | Measure | a | p | w | c' | b | q | v |  | 10 | 25 | 34 | 50 | 66 | 75 | 90 |
| No Results |  |  |  |  |  |  |  |  |  |  |  |  |  |  |  |  |  |
| Notes: Hemi: hemisphere, Measure: (T)hickness or (V)olume, a: parameter relating age group to brain measure, p: parameter relating lifetime exposures (LE) to brain measure, w: interaction parameter of age group and LE in predicting brain measure, c’: parameter relating age group to cognitive measure, b: parameter relating brain measure to cognitive measure, q: parameter relating lifetime exposure (LE) to cognitive measure, v: interaction parameter of LE and brain measure in predicting cognitive measure. The last seven columns are the indirect effects of age group on the cognitive measure via the brain measure probed at percentiles of LE. *significant at *p*(uncorrected) < 0.05. | | | | | | | | | | | | | | | | | |

| Table S9. Speed, Model D |  |  |  |  |  |
| --- | --- | --- | --- | --- | --- |
| Region | Hemi | Measure | a | c' | b |
| Accumbens | L | V | -1.0* | -1.1* | 0.2* |
| Rost. Ant. Cingulate | L | T | -0.9* | -1.0* | 0.2* |
| Trans. Temporal | L | T | -1.0* | -0.9* | 0.3* |
| bankssts | R | T | -0.8* | -1.4* | -0.2* |
| Isthmus of Cingulate | R | T | -0.8* | -1.0* | 0.2* |
| Med. Orbitofrontal | R | T | -0.8* | -1.1* | 0.2 |
| Frontal Pole | R | T | -0.5* | -1.1* | 0.3* |
| Trans. Temporal | R | T | -0.8* | -1.1* | 0.2 |

Notes: Hemi: hemisphere, Measure: (T)hickness or (V)olume, a: parameter relating age group to brain measure, c’: parameter relating age group to cognitive measure, b: parameter relating brain measure to cognitive measure. *significant at *p*(uncorrected) < 0.05.

| Table S10. Fluid Ability, Model A | | | | | | | | | | | | | | | |
| --- | --- | --- | --- | --- | --- | --- | --- | --- | --- | --- | --- | --- | --- | --- | --- |
|  |  |  |  |  |  |  |  |  | Percentiles | | | | | | |
| Region | Hemi | Measure | a | p | w | c' | b |  | 10 | 25 | 34 | 50 | 66 | 75 | 90 |
| Parsorbitalis | L | T | -1.0* | -0.6* | 0.7* | -1.2* | -0.2 |  | 0.323 | 0.275 | 0.234 | 0.161 | 0.101* | 0.072 | 0.046 |
| Isthmus of Cingulate | R | T | -0.8* | -0.1 | 0.5* | -0.9* | 0.2 |  | -0.241* | -0.207 | -0.178 | -0.126 | -0.084 | -0.063 | -0.045 |
| Notes: Hemi: hemisphere, Measure: (T)hickness or (V)olume, a: parameter relating age group to brain measure, p: parameter relating lifetime exposure (LE) to brain measure, w: interaction parameter of age group and LE in predicting brain measure, c’: parameter relating age group to cognitive measure, b: parameter relating brain measure to cognitive measure. The last seven columns are the indirect effects of age group on the cognitive measure via the brain measure probed at percentiles of LE. *significant at *p*(uncorrected) < 0.05. | | | | | | | | | | | | | | | |

| Table S11. Fluid Ability, Model B | | | | | | | | | | | | | | | |
| --- | --- | --- | --- | --- | --- | --- | --- | --- | --- | --- | --- | --- | --- | --- | --- |
|  |  |  |  |  |  |  |  |  | Percentiles | | | | | | |
| Region | Hemi | Measure | a | c' | b | q | v |  | 10 | 25 | 34 | 50 | 66 | 75 | 90 |
| Caudate | L | V | -0.8* | -1.1* | 0 | 0.7* | 0.2* |  | 0.2* | 0.1* | 0.1 | 0 | -0.1 | -0.1 | -0.1 |
| Putamen | L | V | -1.2* | -1.0* | 0.1 | 0.7* | 0.2* |  | 0.2 | 0.1 | 0 | -0.1 | -0.3* | -0.4* | -0.4* |
| Accumbens | L | V | -1.0* | -1.0* | 0.2* | 0.7* | 0.3* |  | 0.2 | 0 | -0.1 | -0.2* | -0.4* | -0.4* | -0.5* |
| Caudate | R | V | -0.9* | -1.1* | 0 | 0.7* | 0.2* |  | 0.2* | 0.1* | 0.1 | 0 | -0.1 | -0.2 | -0.2* |
| Putamen | R | V | -1.1* | -1.1* | 0.1 | 0.7* | 0.3* |  | 0.3 | 0.2 | 0.1 | -0.1 | -0.3* | -0.3* | -0.4* |
| Hippocampus | R | V | -0.7* | -1.1* | 0.1 | 0.7* | 0.2* |  | 0.1 | 0 | 0 | -0.1 | -0.2* | -0.2* | -0.2* |
| Amygdala | R | V | -0.7* | -1.1* | 0.1 | 0.7* | 0.2* |  | 0.1 | 0.1 | 0 | -0.1 | -0.1* | -0.2* | -0.2* |
| Med. Orbitofrontal | L | T | -0.6* | -1.0* | 0.1 | 0.7* | 0.2* |  | 0.1 | 0 | 0 | -0.1 | -0.2* | -0.2* | -0.2* |
| Rost. Ant. Cingulate | L | T | -0.9* | -1.1* | 0 | 0.7* | 0.2* |  | 0.2* | 0.1 | 0.1 | 0 | -0.1 | -0.1 | -0.2 |
| Insula | L | T | -1.0* | -1.2* | 0 | 0.8* | 0.2* |  | 0.3* | 0.2* | 0.1 | 0 | -0.1 | -0.1 | -0.2 |
| Bank of the superior temporal sulcus | R | T | -0.8* | -1.0* | 0.1 | 0.7* | 0.2* |  | 0.1 | 0.1 | 0 | -0.1 | -0.1* | -0.2* | -0.2* |
| Mid. Caudal Frontal | R | T | -0.9* | -1.1* | 0.1 | 0.7* | 0.2* |  | 0.1 | 0.1 | 0 | -0.1 | -0.2* | -0.2* | -0.3* |
| Mid. Temporal | R | T | -1.0* | -1.0* | 0.1 | 0.8* | 0.2* |  | 0.1 | 0.1 | 0 | -0.1 | -0.2 | -0.3* | -0.3* |
| Post. Central | R | T | -0.7* | -1.0* | 0.1 | 0.7* | 0.2* |  | 0.1 | 0 | 0 | -0.1* | -0.2* | -0.2* | -0.3* |
| Sup. Temporal | R | T | -1.1* | -0.9* | 0.2 | 0.7* | 0.2* |  | 0.1 | 0 | -0.1 | -0.2* | -0.3* | -0.4* | -0.4* |
| Insula | R | T | -0.8* | -1.1* | 0 | 0.8* | 0.2* |  | 0.1* | 0.1 | 0 | 0 | -0.1 | -0.1 | -0.2 |
| Notes: Hemi: hemisphere, Measure: (T)hickness or (V)olume, a: parameter relating age group to brain measure, c’: parameter relating age group to cognitive measure, b: parameter relating brain measure to cognitive measure, q: parameter relating lifetime exposure (LE) to cognitive measure, v: interaction parameter of LE and brain measure in predicting cognitive measure. The last seven columns are the indirect effects of age group on the cognitive measure via the brain measure probed at percentiles of LE. *significant at *p*(uncorrected) < 0.05. | | | | | | | | | | | | | | | |

| Table S12. Fluid Ability, Model C | | | | | | | | | | | | | | | | | |
| --- | --- | --- | --- | --- | --- | --- | --- | --- | --- | --- | --- | --- | --- | --- | --- | --- | --- |
|  |  |  |  |  |  |  |  |  |  |  | Percentiles | | | | | | |
| Region | Hemi | Measure | a | p | w | c' | b | q | v |  | 10 | 25 | 34 | 50 | 66 | 75 | 90 |
| Post. Central | R | T | -0.7* | -0.2 | 0.5* | -1.0* | 0.1 | 0.7* | 0.2* |  | 0.166 | 0.046 | -0.031 | -0.109* | -0.117 | -0.102 | -0.078 |
| Notes: Hemi: hemisphere, Measure: (T)hickness or (V)olume, a: parameter relating age group to brain measure, p: parameter relating lifetime exposures (LE) to brain measure, w: interaction parameter of age group and LE in predicting brain measure, c’: parameter relating age group to cognitive measure, b: parameter relating brain measure to cognitive measure, q: parameter relating lifetime exposure (LE) to cognitive measure, v: interaction parameter of LE and brain measure in predicting cognitive measure. The last seven columns are the indirect effects of age group on the cognitive measure via the brain measure probed at percentiles of LE. *significant at *p*(uncorrected) < 0.05. | | | | | | | | | | | | | | | | | |

| Table S13. Fluid Ability, Model D | | | | | |
| --- | --- | --- | --- | --- | --- |
| Region | Hemi | Measure | a | c' | b |
| Accumbens | L | V | -1.0* | -0.7* | 0.4* |
| VentralDC | L | V | -0.7* | -0.9* | 0.3* |
| Lingual | L | T | -1.0* | -0.8* | 0.2 |
| Parahippocampal | L | T | -0.5* | -1.1* | -0.2* |
| Post. Central | L | T | -0.8* | -0.8* | 0.2 |
| Trans. Temporal | L | T | -1.0* | -0.6* | 0.4* |
| Lingual | R | T | -1.0* | -0.8* | 0.2* |
| Pericalcarine | R | T | -0.7* | -1.2* | -0.2 |
| Post. Central | R | T | -0.7* | -0.9* | 0.2 |
| Trans. Temporal | R | T | -0.8* | -0.7* | 0.4* |
| Notes: Hemi: hemisphere, Measure: (T)hickness or (V)olume, a: parameter relating age group to brain measure, c’: parameter relating age group to cognitive measure, b: parameter relating brain measure to cognitive measure. *significant at p(uncorrected) < 0.05. | | | | | |
